# Association between Use of HMG CoA Reductase Inhibitors and Mortality in HIV-Infected Patients

**DOI:** 10.1371/journal.pone.0021843

**Published:** 2011-07-12

**Authors:** Richard D. Moore, John G. Bartlett, Joel E. Gallant

**Affiliations:** Department of Medicine, Johns Hopkins University School of Medicine, Baltimore, Maryland, United States of America; University of Cape Town, South Africa

## Abstract

**Introduction:**

HIV infection is a disease associated with chronic inflammation and immune activation. Antiretroviral therapy reduces inflammation, but not to levels in comparable HIV-negative individuals. The HMG-coenzyme A reductase inhibitors (statins) inhibit several pro-inflammatory processes and suppress immune activation, and are a logical therapy to assess for a possible salutary effect on HIV disease progression and outcomes.

**Methods:**

Eligible patients were patients enrolled in the Johns Hopkins HIV Clinical Cohort who achieved virologic suppression within 180 days of starting a new highly active antiretroviral therapy (HAART) regimen after January 1, 1998. Assessment was continued until death in patients who maintained a virologic suppression, with right-censoring of their follow-up time if they had an HIV RNA > 500 copies/ml. Cox proportional hazards regression was used to assess statin use as a time-varying covariate, as well as other demographic and clinical factors.

**Results:**

A total of 1538 HIV-infected patients fulfilled eligibility criteria, of whom 238 (15.5%) received a statin while taking HAART. There were 85 deaths (7 in statin users, 78 in non-users). By multivariate Cox regression, statin use was associated with a relative hazard of 0.33 (95% CI: 0.14, 0.76; P = 0.009) after adjusting for CD4, HIV-1 RNA, hemoglobin and cholesterol levels at the start of HAART, age, race, HIV risk group, prior use of ART, year of HAART start, NNRTI vs. PI-based ART, prior AIDS-defining illness, and viral hepatitis coinfection. Malignancy, non-AIDS-defining infection and liver failure were particularly prominent causes of death.

**Discussion:**

Statin use was associated with significantly lower hazard of dying in these HIV-infected patients who were being effectively treated with HAART as determined by virologic suppression. Our results suggest the need for confirmation in other observational cohorts, and if confirmed, the need for a clinical trial of statin use in HIV infection.

## Introduction

It is now well-recognized that HIV infection causes chronic inflammation and immune activation. Untreated HIV infection is associated with persistently high levels of pro-inflammatory cytokines such as IL-1, IL-6, and TNF-alpha, coagulation biomarkers and acute phase proteins such as fibrinogen, D-dimer and high-sensitivity C-reactive protein [1], [2]. These markers of inflammation decline with the use of effective antiretroviral therapy (ART), suggesting that active HIV replication is responsible for this inflammatory response. However, many of these inflammatory markers remain elevated despite suppression of HIV replication with ART [3]. This may be due to low-level ongoing HIV replication, other pathogens such as cytomegalovirus, and/or translocation of gut-associated bacteria releasing lipopolysaccharide (LPS) due to damage to gastrointestinal lymphoid tissue and the intestinal lining [4], [5]. Immune activation is also triggered by pro-inflammatory cytokines, and their continuous activation, together with thymic dysfunction and reduced T cell generative potential, appears to accelerate the development of early immunosenescence [2], [6], [7]. Chronic inflammation and immune activation accelerate organ damage to a variety of organ systems [8], [9]. Cardiovascular disease, malignancy, chronic kidney and liver disease all appear to be occurring earlier and more frequently in HIV-infected individuals, and have each been associated with this process [10]–[13].

Given this scenario, it is possible that anti-inflammatory treatments might improve HIV outcomes. ART is not sufficient, and other measures that reduce inflammation might provide additional benefit. The 3-hydroxy-3-methylglutaryl-coenzyme A reductase inhibitors (“statins”) are a class of drugs that have been found to inhibit several pro-inflammatory processes independent of their cholesterol-lowering effects. They decrease pro-inflammatory cytokine levels and acute phase proteins, and also appear to repress the activation of T lymphocytes [14]–[18]. In addition to their beneficial effect on atherosclerosis, there is evidence that statins may have other therapeutic benefits unrelated to anti-inflammatory effects, such as a reduced risk of malignancy and other non-cardiovascular disease complications including reduced mortality from pneumonia, sepsis and influenza [19]–[27].

The relatively widespread use of statins in HIV-infected individuals make them a logical drug class to examine in an observational cohort of HIV-infected patients receiving ART. Since ART is the cornerstone of HIV therapy, our analysis focused on the association of statin use with clinical outcomes in patients who maintained a full virologic suppression on ART.

## Methods

This study was approved by the Johns Hopkins University School of Medicine institutional review board. All subjects gave written informed consent to have their clinical data collected longitudinally for research purposes. The clinical investigation was conducted according to the principles expressed in the Declaration of Helsinki.

This analysis was conducted using data from patients in the Johns Hopkins HIV Clinical Cohort. Briefly, this is a longitudinal cohort study that enrolls patients who present for HIV care at the Johns Hopkins HIV/AIDS Clinics. The refusal rate for participation is < 0.5%. Starting with their first clinic visit, demographic data, all medication use, clinical diagnoses (HIV and non-HIV), and comprehensive laboratory data are collected [28]).

This analysis examined survival after the initiation of a new HAART regimen after cohort enrollment from January 1, 1998 through December 31, 2009. We further limited the analysis to patients who achieved suppression of HIV-1 RNA level to undetectable levels within 6 months of starting HAART (< =  400 copies/ml by first generation PCR assay or <50 copies/ml by ultrasensitive PCR assay). HAART was defined as use of a new non-nucleoside reverse transcriptase inhibitor (NNRTI), protease inhibitor (PI) or integrase strand transfer inhibitor (InSTI) with two nucleoside analog reverse transcriptase inhibitors (NRTIs), or use of an NNRTI plus PI and/or InSTI. The time origin was the day of initiation of the new HAART regimen, and patients had to have received at least 90 days of this regimen to qualify for analysis. Statin use was coded as present if the statin was being received by the patient at the time of, or after initiation of HAART. A patient had to receive a statin for at least 30 days to be considered as a statin user. Use of antiretroviral agents and statins was determined based on prescribing, not filling of prescriptions, so adherence was not directly assessed.

Mortality data, including date and cause of death, are obtained by regular assessment of National Death Index and the National Social Security Death Index. In addition, we routinely determine deaths reported in the medical records from our institutions and other facilities, by telephone or other communication from relatives, friends, and other medical providers.

Cox proportional hazards regression was used to examine the association of statin use with death. For this analysis, the initiation of the HAART regimen was the time origin. The patient contributed follow-up time only as long as s/he maintained a suppressed viral load. Follow-up was right-censored if the patient developed a viral load of greater than 500 copies/ml, or 90 days after the last undetectable viral load measurement if no further values were obtained. We examined statin use as a time-varying variable, since patients who received statins were not always receiving them at the time that the HAART regimen was started. Analyzing statin use as time-varying minimizes immortal-time bias (i.e., the patient must survive to receive the drug) that would occur if statin use was analyzed simply as a fixed covariate. The risk sets include whether a statin is being used or not at that particular time after starting HAART, irrespective of whether the patient received a statin at some other time during follow-up. Statin use was also lagged by 90 days if it was discontinued so that a death would attributed to a statin user if the statin had been used at least 90 days prior to the event.

Other variables that were examined included age, sex, race, HIV transmission risk group, hemoglobin level at HAART initiation, CD4+ cell count at HAART initiation, HIV-1 RNA level at HAART initiation, specific HAART anchor drug (NNRTI, PI, InSTI), calendar date of initiation of the HAART regimen (< 2004 vs. ≥ 2004), prior ART use before starting this HAART regimen prior AIDS-defining illness (ADI), and a random total cholesterol level at HAART initiation. We compared these patient characteristics of the sample by receipt of a statin using the Wilcoxon rank-sum test for continuous variables or the chi-squared test for categorical variables. Each of these variables were also analyzed as covariates in a multivariate Cox regression.

Finally, we conducted a similar analysis to that described above for the use of antihypertensive therapy. Since antihypertensive therapy is often used in patients who are prescribed statins, we wished to see if this therapy was associated with survival as a method to assess for potential bias in the prescribing of these drugs to individuals who were believed by their providers to be more adherent or otherwise better candidates for cardiovascular risk intervention.

## Results

We analyzed the data from 1538 HIV-infected patients who achieved virologic suppression within 6 months of starting HAART and who subsequently maintained suppression until death or until their data were censored. The median time to censoring was 570 days (interquartile range: 268–1286 days), with no significant difference in time to censoring between those receiving and not receiving statins. The demographic and clinical characteristics of the sample are shown in Table 1. The sample characteristics reflected the demography of HIV-infection in Maryland [29]. A total of 238 (15.5%) received a statin (69% atorvastatin, 24% pravastatin, 7% rosuvastatin). Patients who received statins were older and more likely to be white, and men who have sex with men (MSM). They also had higher CD4+ cell count and hemoglobin levels at baseline, and were more likely to have previously received ART and to have had a prior ADI. Statin users were more likely to have started HAART in the early part of the decade than non-statin users. Statins were being used at the time of HAART initiation in 122 (51.2%) of the patients who received a statin. The median time on statin therapy was 745 days.

**Table 1:** Characteristics of the study population.

		Total	Statin Use	No Statin Use
Category	Subcategory	N = 1538	N = 238	N = 1300
Age (median years)		43 (36–49)	46 (40–53)	42 (36–47)**
Sex	Male	1034 (67.2%)	172 (72.3%)	862 (66.3%)
	Female	504 (32.7%)	66 (27.7%)	438 (33.7%)
Race	Black	1112 (72.3%)	138 (58.0%)	974 (74.9%)**
	White	292 (22.5%)	92 (38.7%)	292 (22.5%)**
	Other	40 (2.7%)	8 (3.4%)	34 (2.6%)
HIV Risk Group^1^	IDU	551 (34.3%)	48 (20.2%)	503 (38.7%)**
	MSM	445 (28.9%)	91 (38.2%)	354(27.2%)**
	Heterosexual Contact	791 (51.7%)	106 (44.5%)	685 (52.7%)*
CD4 (median cells/mm^3^)		225 (80–358)	270 (153–461)	200 (70–339)**
HIV-1 RNA (median copies/ml)		36,186 (1,633–145,950)	11,149 (88–126,816)	39,879 (3,252–163,006)**
Hemoglobin (median g/dL)		12.7 (11.3–14.1)	13.2 (12.0–14.6)	12.7 (11.3–14.0)**
Total cholesterol (median mg/dL)		166 (141–194)	196 (168–238)	160 (134–188)**
Antihypertensive Use		407 (29.3%)	114 (46.3%)	293 (25.6%)**
Prior ART		933 (60.7%)	176 (73.9%)	757 (58.2%)**
Prior ADI		720 (46.8%)	132 (55.4%)	588 (45.2%)**
Viral hepatitis C co-infection		537 (34.9%)	61 (25.6%)	476 (36.6%)**
Year HAART started	< 2004	875 (56.9%)	159 (66.8%)	716 (55.1%)**
	≥2004	663 (43.1%)	79 (33.2%)	584 (44.9%)

*0.01 < p-value < 0.5.

**p-value < 0.01.

1 Categories are not mutually exclusive.

There were 85 deaths (7 on statins, 78 not on statins). By multivariate Cox regression, statin use was associated with a relative hazard (RH) of 0.33 (95% CI: 0.14, 0.76, p = 0.009), adjusting for CD4, HIV-1 RNA, hemoglobin and cholesterol levels at the start of HAART, age, race, HIV risk group, prior use of ART, year of HAART start, NNRTI vs. PI-based ART, prior AIDS-defining illness, and viral hepatitis coinfection (Table 2). Of these other characteristics, those that were also associated with an increased risk of death in this multivariate analysis were a low baseline hemoglobin level, older age, injection drug use (IDU), and a prior ADI. Interactions between statin use and IDU, heterosexual risk, sex, age > 50 years, race and prior ADI were all tested, and none were significant, indicating that there is no significant difference in the association between statin use and survival by the individual categories of these variables. Statin use was tested for and met the proportionality assumption for the Cox proportional hazards model.

**Table 2:** Multivariate Cox Proportional Hazards Regression of statin use and other characteristics with survival.

Category	Subcategory	Relative Hazard (95% CI)	P-Value
Statin Use		0.33 (0.14,0.76)	0.009
Age (median years)		1.07 (1.05,1.10)	<0.0001
Race	Black	0.82 (0.47, 1.46)	0.51
	Others	1.0 (reference)	
HIV Risk Group	IDU	2.30 (1.30, 4.07)	0.004
	Heterosexual	1.50 (0.96, 2.35)	0.08
	MSM	1.0 (reference)	
CD4+ at HAART start (per 100 cell/mm^3^ higher increments)		0.96 (0.84, 1.09)	0.52
HIV-1 RNA at HAART start (per log_10_ higher increments)		0.96 (0.79, 1.18)	0.16
Hemoglobin at HAART start (per g/dL higher increments)		0.80 (0.71, 0.90)	0.0003
Total Cholesterol at HAART start (per 10 mg/dL higher increments)		0.98 (0.93, 1.03	0.36
Year HAART started	< = 2004	1.20 (0.74, 2.06)	0.50
	> 2004	1.0 (reference)	
HAART Drug	NNRTI	1.23 (0.59, 1.52)	0.42
	Others	1.0 (reference)	
Prior ART		1.37 (0.82, 2.31)	0.23
Prior ADI		2.24 (1.39, 3.60)	0.001
Viral Hepatitis C Co-infection		1.07 (0.62, 1.84)	0.81

*Male vs. female sex could not be analyzed independently because of collinearity with the MSM risk group.

(Multivariate adjusted association of Statin use and each of the other variables)1 categroies are0 mg/dL increase)g/dL increase).

The causes of death are shown in Table 3. Malignancy, non-AIDS-defining infection and liver failure were particularly prominent. There was no proportional difference in death caused by a cardiovascular disease among statin users compared to non-users, however the small number of deaths in statin users makes meaningful statistical comparisons difficult. In addition, there were 15 deaths of unknown cause, some of which could have been due to an unwitnessed cardiovascular event.

**Table 3:** Causes of death.

Cause	Statin Use (#)	No Statin Use (#)
Malignancy	2	14
Infection (non-ADI)	2	12
Metabolic Complications of diabetes		1
Neuromuscular Disease Complications		2
End Stage or Acute Liver Failure		11
End Stage or Acute Renal Failure		6
Cardiovascular	2	10
Pulmonary Embolus		2
Trauma		3
Substance Overdose		3
Unknown*	1	14

*Date of death confirmed: 6 were found dead at home but the cause was unknown; 9 others were confirmed dead but no further documentation could be obtained.

A total of 407 of the patients received antihypertensive therapy. There was substantial overlap among users of statins and antihypertensives, with 46% of those on statins also receiving antihypertensive therapy. An analysis of antihypertensive therapy, done similarly to the statin use analysis, showed no association with survival (RH = 1.11; 95% CI: 0.70, 1.78, p = 0.64).

## Discussion

We found that statin use was associated with a significantly decreased hazard of dying. Statins have anti-inflammatory and cellular immunological effects [14]–[18]. There is some evidence of inhibition of HIV replication by statins in vitro [30], [31], but this effect was not seen clinically [15], [32]. Atorvastatin was the most commonly used statin, but we are unable to assess the association between mortality and an individual type of statin due to the small number of deaths in the statin using patients.

It is notable that a lower baseline hemoglobin was also strongly associated with survival in these patients, as others have also found [33]–[34]. “Anemia of chronic disease” is an anemia of chronic inflammation, regulated by the peptide hormone hepcidin, whose expression is increased during inflammation, infection, and iron overload [35]. Arguably, anemia is a reasonable measure of chronic inflammation when more specific inflammatory biomarkers such are not routinely measured in clinical practice. Anemia has been shown to be associated with survival in HIV-infected persons. The strong association between anemia and survival in our patients whose viral loads were suppressed suggests an independent association between ongoing inflammation and mortality. The small number of deaths among our patients who received statins did not allow us to analyze whether there was a differential association between statin use and death in those with low versus a high hemoglobin levels, but there was not a significant interaction between statin use and hemoglobin level and survival. Since other inflammatory biomarkers or cellular markers of immune activation were almost never measured in this cohort as part of clinical care, we are unable to examine the association between these other biomarkers, statin use and mortality. Other prognostic variables included older age and non-MSM HIV transmission risk categories. Since both IDU and heterosexual contact were associated with a higher risk of death than MSM, we assessed for interactions between statin use and both IDU and heterosexual contact. These interactions were not significant, indicating that the association between statin use and death was similar in each HIV transmission risk group. The baseline CD4+ cell count at the start of therapy was not associated with mortality in the multivariate adjusted analysis, suggesting that this may be a less important prognostic variable among our patients who maintain virologic suppression for causes of death that were not AIDS-defining illnesses.

No observational analysis can provide definitive evidence for the efficacy of drug therapy, since selection bias can affect the associations found. Since statins could have been selectively prescribed to individuals thought to be more adherent or at lower risk of dying, we adjusted for both other prognostic variables (variables associated with death) and several variables such as MSM that were associated with use of statins. There were no significant interactions between statin use and survival and HIV risk group or several other variables. We also analyzed the association of antihypertensive drugs, another class of therapy used to manage cardiovascular disease risk, and that also tends to also be used in patients who receive statins (60% overlap in our population). There was no association between antihypertensive therapy and survival. We were unable to assess other anti-inflammatory and immunosuppressive drugs either because of infrequent use (e.g., prednisone, cyclosporin), or over the counter use that we did not capture (e.g. aspirin, NSAIDS). Since all of the patients in this analysis maintained suppressed viral loads, we believe that differential adherence to therapy was less likely to be a significant issue. Nevertheless, it is certainly possible that statins were used selectively in patients with a better survival prognosis and that there are unmeasured confounders that would explain the association we found. Even if statin use is clinically causing a reduced mortality, it could be due to reasons other than an effect on inflammation and immune activation.

In summary, we found that patients who maintained virologic suppression on effective HAART appeared to derive additional survival benefit from the use of a statin. If additional observational data support this finding, a randomized clinical trial would be warranted to confirm this association.
